# A twelve days’ male baby with clinodactyly: a rare clinical image

**DOI:** 10.11604/pamj.2023.45.178.41119

**Published:** 2023-08-23

**Authors:** Sangita Nade, Bibin Kurian

**Affiliations:** 1 Department of Child Health Nursing, Smt Radhikabai Meghe Memorial College of Nursing, Datta Meghe Institute of Higher Education & Research (DM) Sawangi Meghe, Wardha, India

**Keywords:** Clinodactyly, anomaly, arch, palate

## Image in medicine

Clinodactyly is defined as a congenital curvature of a digit distal to the metacarpal phalangeal joint in the coronal plane. Curvatures with an angular deviation of fewer than 10 degrees can be seen as a normal physiologic variant. Clinodactyly is defined when the coronal angulation of the affected digit is greater than 10 degrees. The curvature arises due to the abnormal trapezoidal or triangular shape of one or more phalanges, which leads to malalignment of the associated interphalangeal joint. This abnormal shape leads to asymmetric longitudinal growth in a direction deviated from the normal longitudinal axis of the finger resulting in the visible curvature of the digit. The patient b/o Pooja Shinde Fch day of life 12 Referral from Rural Hospital, Digras I/V/O feeding difficulty. The baby was admitted to NICU private hospital after birth for a week cry /lscs/birth weight 3kg/ current weight 2.4 kg was on O_2_ support for 3 days on intragastric feed since birth. On admission RBS-101 mg/dl, SPO_2_-94 on room air, heart rate-160, high arch palate clinodactyly. Kept NBM with maintenance fluid with antibiotics. Mother has a history of PIH with 1 abortion (rupture of ectopic pregnancy).

**Figure 1:**
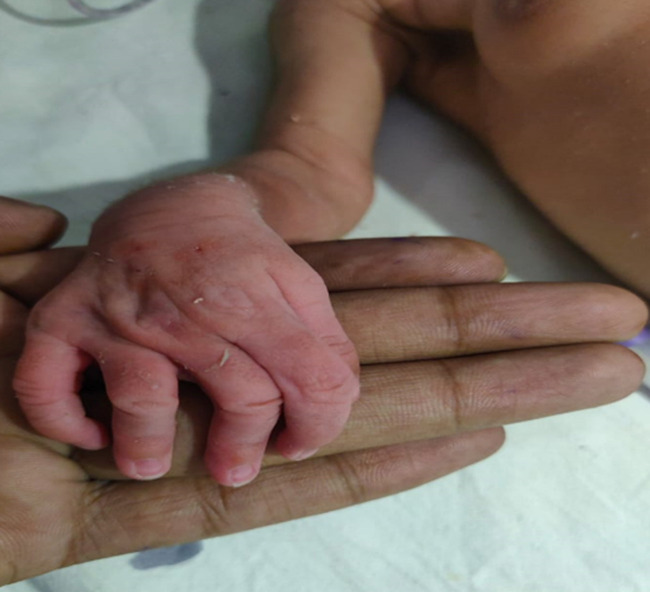
clinodactyly

